# A Human Corneal Epithelial Cell Line Model for Limbal Stem Cell Biology and Limbal Immunobiology

**DOI:** 10.5966/sctm.2016-0175

**Published:** 2016-10-14

**Authors:** Bakiah Shaharuddin, Sajjad Ahmad, Nani Md Latar, Simi Ali, Annette Meeson

**Affiliations:** ^1^ Institute of Genetic Medicine, Newcastle University, Newcastle Upon‐Tyne, United Kingdom; ^2^ Advanced Medical and Dental Institute, Universiti Sains Malaysia, Pulau Pinang, Malaysia; ^3^ St. Paul's Eye Unit, Royal Liverpool University Hospital, Liverpool, United Kingdom; ^4^ Department of Eye and Vision Sciences, Institute of Ageing and Chronic Disease, University of Liverpool, Liverpool, United Kingdom; ^5^ Department of Surgery, Faculty of Medicine, Universiti Kebangsaan, Malaysia Medical Centre, Kuala Lumpur, Malaysia; ^6^ Institute of Cellular Medicine, Newcastle University, Newcastle Upon‐Tyne, United Kingdom

**Keywords:** Limbal stem cell, Immunobiology, Side population, ABCB5, CXCR4

## Abstract

Limbal stem cell (LSC) deficiency is a visually debilitating condition caused by abnormal maintenance of LSCs. It is treated by transplantation of donor‐derived limbal epithelial cells (LECs), the success of which depends on the presence and quality of LSCs within the transplant. Understanding the immunobiological responses of these cells within the transplants could improve cell engraftment and survival. However, human corneal rings used as a source of LSCs are not always readily available for research purposes. As an alternative, we hypothesized that a human telomerase‐immortalized corneal epithelial cell (HTCEC) line could be used as a model for studying LSC immunobiology. HTCEC constitutively expressed human leukocyte antigen (HLA) class I but not class II molecules. However, when stimulated by interferon‐γ, HTCECs then expressed HLA class II antigens. Some HTCECs were also migratory in response to CXCL12 and expressed stem cell markers, Nanog, Oct4, and Sox2. In addition because both HTCECs and LECs contain side population (SP) cells, which are an enriched LSC population, we used these SP cells to show that some HTCEC SP cells coexpressed ABCG2 and ABCB5. HTCEC SP and non‐side population (NSP) cells also expressed CXCR4, but the SP cells expressed higher levels. Both were capable of colony formation, but the NSP colonies were smaller and contained fewer cells. In addition, HTCECs expressed ΔNp63α. These results suggest the HTCEC line is a useful model for further understanding LSC biology by using an in vitro approach without reliance on a supply of human tissue. Stem Cells Translational Medicine *2017;6:761–766*

**Significance Statement**

Limbal stem cell deficiency is a painful eye condition caused by abnormal maintenance of limbal stem cells. It is treated by transplantation of limbal epithelial cells derived from human tissue. The success of this treatment depends of the quality of the cells transplanted; however, some transplants fail. Understanding more about the immunobiology of these cells within the transplants could improve the outcomes. However, the human tissue needed as a supply of stem cells for this research is not readily available. As an alternative, a human telomerase‐immortalized corneal epithelial cell line may be used. This study shows that this cell line contains limbal stem cells. Moreover, these cells have characteristics and immunobiological functions similar to those of tissue‐derived limbal cells. These results suggest that this cell line is a useful model for improving the understanding of limbal stem cell biology.

## Introduction

Limbal stem cell deficiency (LSCD) is a debilitating eye condition in which, after damage to the corneal epithelium, the cornea fails to regenerate. This failure is due to the loss of limbal stem cells (LSCs) and ultimately leads to chronic ocular pain and loss of vision. Tissue availability, small cell yields, and patient‐to‐patient sample variability can limit limbal studies. Therefore, we proposed the use of a human telomerase‐immortalized corneal epithelial cell (HTCEC) line as a model for studying LSC biology. HTCECs were originally derived by forced expression of human telomerase reverse transcriptase in human epithelial cells and, importantly, have been shown to have stratification and differentiation potential similar to those of normal human epithelial cells in vitro [1]. We examined HTCECs for stem cell properties and biological and immunological functions. In addition, we further characterized the HTCEC side population (SP). Limbal SP cells (LSPs) have features consistent with those of stem cells [2, 3].

## Materials and Methods

Human tissue use was conducted with ethical approval from the Ethics Committee, Newcastle University, United Kingdom, and in accordance with the ethical principles of the Declaration of Helsinki.

### Limbal Epithelial Cell Culture and Side Population Assay

Both were isolated as described previously [3]. In brief, LECs were isolated from tissue by using serial trypsinization, then plated onto irradiated 3T3 fibroblasts and harvested for analysis at day 10. Both LECs and HTCECs were stained with 3 µg/ml Hoechst 33342 dye for 45 minutes before fluorescence‐activated cell sorting (FACS) analysis.

### HTCECs

HTCECs were a gift from Professor Kao, University of Cincinnati, Cincinnati, Ohio, and were originally derived by Professor Jester, University of California, Irvine. HTCECs were propagated as described previously [4].

### Immunocytochemistry

Immunohistochemistry (ICC) was performed as described previously [4, 5]. Briefly, cells for ICC were fixed with cold methanol, washed, and permeabilized, and nonspecific binding sites were blocked by incubation in appropriate blocking serum for 30 minutes. Cells were then incubated with primary antibodies, followed by appropriate fluorophore conjugated secondary antibodies. Details of antibodies used are provided in supplemental online Table 1.

### Semi‐Quantitative Polymerase Chain Reaction

Semi‐quantitative polymerase chain reaction was performed as described previously [4, 5]. Oligonucleotide primers and amplification conditions are presented in supplemental online Table 2 and in Di Iorio et al. [6].

### FACS

For direct immunofluorescence, 2 × 10^5^ cells in 100‐µl cells suspensions were stained with 5 µl primary antibody for 1 hour. Cells were then washed and resuspended in 200 µl buffer solution and analyzed by using FACS. For indirect immunofluorescence, 2 × 10^5^ cells were stained with 5 µl primary antibody in 100‐µl cells suspensions for 1 hour. Cells were then washed and incubated in appropriate fluorophore conjugated secondary 1:25 dilution for 30 minutes, washed again, and resuspended in 200 µl buffer solution and analyzed by using FACS Canto and FACS Diva software [BD Biosciences, Oxford, UK, http://www.bdbiosciences.com]. Antibodies used for FACS analysis are provided in supplemental online Table 1.

### Transwell Migration Analysis

A total of 1 × 10^5^ HTCECs were resuspended in 300 µl defined keratinocyte serum‐free medium (Thermo Fisher Scientific Life Sciences, Waltham, MA, http://www.thermofisher.com) and added to the upper chamber of a 5‐µm‐pore‐diameter 24‐well format transwell chamber. The lower chamber contained 200 µl media without cells but supplemented with CXCL12 at 300 ng/ml (control was 0 ng/ml), and cells were cultured for 5 hours under standard tissue culture conditions. After this, the filters were removed and stained with hematoxylin. Migrant cells were counted (5 randomly selected high‐power fields per well at original magnification, ×20).

### Microscopy and Imaging

All images were taken by using a Nikon Digital Sight‐DSFi1 camera and Nikon NIS‐Elements D software (Nikon Metrology UK Ltd., Derby, UK, http://www.nikonmetrology.com) and were collated by using Adobe Photoshop (Adobe Systems, San Jose, CA, http://www.adobe.com). For fluorescence images, an Axioplan F system was used, and images were processed by using Axio‐Vision40 software (Zeiss, Cambridge, UK, http://www.zeiss.co.uk).

### Human Leukocyte Antigen Typing and HLA Expression in HTCECs

Human leukocyte antigen (HLA) typing was outsourced to National Health Service Blood and Tissue Bank (Newcastle, UK), courtesy of Dr. Carter. To examine HLA expression in HTCECs, cells in culture were treated with interferon (IFN)‐γ1b (Miltenyi Biotec, Bisley, UK, https://www.miltenyibiotec.com) alone, recombinant tumor necrosis factor (TNF)‐α (R&D Systems, Minneapolis, MN, https://www.rndsystems.com) alone or a combination of both, at a preoptimized concentration of 10 ng/ml for 3 days. Samples were then prepared for FACS analysis, as described previously [4, 5]. For HLA expression in unstimulated HTCECs, the negative control was unstained cells; for stimulated cells, the negative control was unstimulated cells.

### Statistical Analysis

Quantitative data were analyzed for comparison between two groups by using an independent *t* test. Results with *p* values <.05% were considered statistically significant.

## Results

### Stem Cell and Limbal Markers in HTCECs and LECs

**Figure 1:**
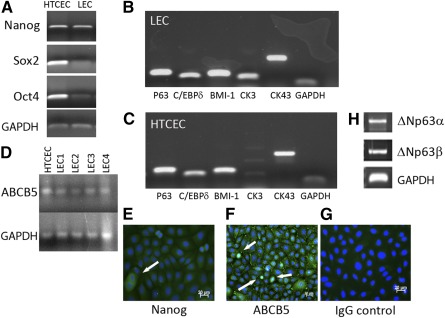
HTCECs and limbal epithelial cells express stem cell and limbal markers. Representative images of results of polymerase chain reaction (PCR) analysis of HTCECs and LECs for mRNA expression of common stem cell markers (note both populations express all three genes) **(A)** and limbal stem cell markers, LECs **(B)** and HTCECs **(C)** (*n* = 5). Lane 1, p63 (143 base pairs [bp]); lane 2, C/EBPδ (111 bp); lane 3, BMI‐1 (132 bp); lane 4, CK3 (125 bp); lane 5, CK43 (249 bp); lane 6, GAPDH (100 bp). HTCECs and LECs (four different primary derived donor LECs) also express ABCB5 mRNA **(D)**. GAPDH was used as loading control throughout. Representative images of immunohistochemistry of analysis of HTCECs show that some cells expressed Nanog **(E)** and ABCB5 **(F)** (positive cells indicated by arrows). **(G)** IgG‐only negative control shows no staining in HTCECs (*n* = 3). Blue, 4′,6‐diamidino‐2‐phenylindole; green, fluorescein isothiocyanate‐conjugated secondary antibody). Results of PCR analysis for ΔNp63 isoforms (H), HTCEC express the α isoform (1388 bp) and the β isoform (1374 bp). Abbreviations: CK3, cytokeratin 3; CK43, connexin 43; HTCEC, human telomerase‐immortalized corneal epithelial cell; GAPDH, glyceraldehyde 3‐phosphate dehydrogenase; HTCEC, human telomerase‐immortalized corneal epithelial cell; LEC, limbal epithelial cell.

mRNA analysis of HTCECs and LECs showed that they expressed the stem cell markers Nanog, Oct4, and Sox2 and the limbal markers P63, C/EBPδ, BMI‐1, cytokeratin 3 (CK3), connexin43, and ABCB5 (Fig. 1A–1D). ICC analysis of HTCECs showed that some cells expressed Nanog and ABCB5 (Fig. 1E, 1F), with no staining in controls (G). mRNA analysis of HTCECs for ΔNp63 isoforms showed that they expressed α and β (H) but not γ (data not shown).

**Figure 2:**
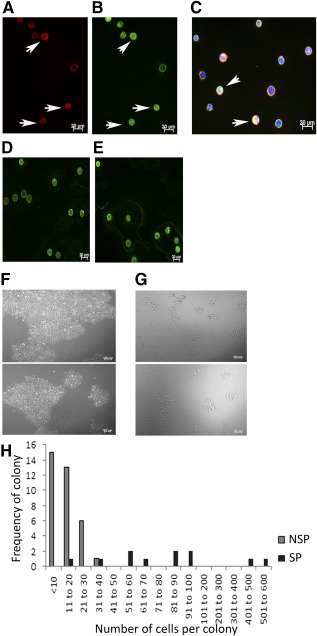
Human telomerase‐immortalized corneal epithelial cell (HTCEC) SP cells express ABCG2 and ABCB5. Representative images of immunohistochemistry analysis of ABCB5 and ABCG2 expression (*n* = 3). Expression of ABCG2 **(A)** and ABCB5 **(B)** in SP cells. Arrows indicate positive cells. **(C):** Image overlay of ABCG2‐ and ABCB5‐stained SP cells shows that some cells express both transporters (indicated by arrows). ABCB5 expression in SP **(D)** and NSP **(E)** cells from the same cell preparation. Green, fluorescein isothiocyanate‐conjugated secondary anti‐mouse antibody; red, rhodamine‐conjugated anti‐rabbit secondary antibody; blue, 4′,6‐diamidino‐2‐phenylindole; scale bars = 20 µm. HTCEC SP and NSP have colony‐forming ability. Phase contrast images show colony formation of SP and NSP‐sorted HTCEC on day 5 of culture **(F)** SP and **(G)** NSP. **(H)**: Number of cells per colony plotted against cell count in SP and NSP cells in HTCEC; difference in the cell number per colony between the SP and NSP cell fractions was significant at *p* = .010. Note that the NSP cells form more colonies but they contain fewer cells. Scale bar = 100 µm. Abbreviations: NSP, non‐side population; SP, side population.

HTCEC SP and NSP both expressed ABCG2 and ABCB5; some cells showed coexpression of both (Fig. 2A–2C). Quantification of ABCB5 expression in both SP and NSP cells isolated from the same cell preparation (Fig. 2D, 2E) showed that the mean signal intensity of ABCB5 expression in SP was 26.35 ± 8.70 and that in NSP was 24.17 ± 8.07. The mean difference between the two groups was significantly higher in SP than NSP (*p =* .02) (supplemental online Table 3).

### Colony‐Forming Analysis of HTCEC SP and NSP

HTCEC SP and NSP both formed colonies (Fig. 2F, 2G), but the NSP colonies were smaller and contained fewer cells. The difference in the cell number per colony between the SP and NSP cell fractions was significant (*p* = .01) (Fig. 2H).

### Chemotactic Potential of HTCECs

**Figure 3:**
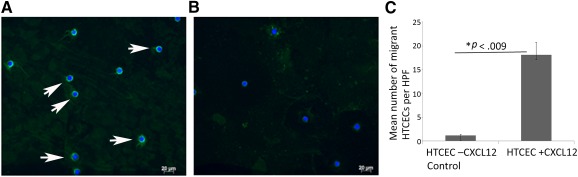
HTCECs express CXCR4 and migrate in response to CXCL12. Representative images of immunohistochemistry analysis for CXCR4 expression in HTCECs (*n* = 3). Expression of CXCR4 in HTCEC side population **(A)** and non‐side population **(B).** White arrows, positive cells; blue, 4′,6‐diamidino‐2‐phenylindole; green, fluorescein isothiocyanate‐conjugated secondary anti‐mouse antibody; scale bars = 20 µm). **(C):** Mean number of migrant cells per HPF from three biological replicates for CXCL12‐mediated migration unsorted HTCECs in comparison with background migration (control). Treatment with CXCL12 ligand was at 300 nM for 5 hours. ∗, Difference in mean values between control and CXCL12‐treated group. Abbreviation: HPF, high‐power field; HTCEC, human telomerase‐immortalized corneal epithelial cell.

ICC analysis (Fig. 3A, 3B) and measurement of mean fluorescent intensity (supplemental online Table 3) for CXCR4 expression showed that both HTCEC SP and NSP expressed CXCR4. However, the NSP had a lower level of CXCR4 expression compared with the SP.

To examine CXCL12‐mediated cellular migration, chemotaxis experiments were performed. The same numbers of HTCECs, but without addition of CXCL12 in the media, were used as control (background migration). After stimulation with 300 nM CXCL12 for 5 hours, we found that HTCECs were migratory in response to CXCL12. The difference in the means of migrant cells per high‐power fields for HTCECs in comparison with background migration (media without CXCL12) were statistically significant (*p* = .009) (Fig. 3C).

### HLA Typing and HLA Expression in HTCECs

**Figure 4:**
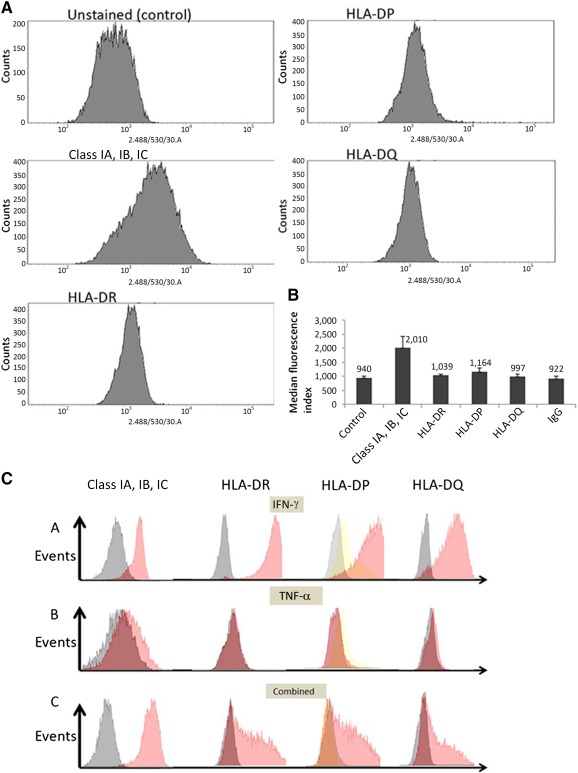
Fluorescence‐activated cell sorting (FACS) analysis of HLA expression in human telomerase‐immortalized corneal epithelial cells (HTCECs) of unstimulated and stimulated cell populations. **(A):** Histograms showing unstained population (control) and cell populations stained with HLA class I A, IB, and IC and Class II antibodies. **(B):** Median fluorescence index of HLA expression for control and stained populations without cytokine stimulation (*n* = 3). Mean median fluorescence index for class I was significantly different than for control but not for other class II molecules. **(C):** Representative FACS histograms out of three replicates showing HLA expression of class I and class II antigens in HTCECs after stimulation with IFN‐γ, TNF‐α, and combined stimulation of both (10 ng/ml, 3 days). Cells were stained with fluorescein isothiocyanate‐conjugated class I, HLA‐DR, HLA‐DQ antibodies, and HLA‐DP. Negative control for HLA‐DP was secondary IgG only. Gray, unstimulated cells; red, stimulated cells; yellow, IgG‐only stimulated cells. Abbreviations: HLA, human leukocyte antigen; IFN, interferon; TNF, tumor necrosis factor.

HLA typing showed that HTCECs expressed both class IA, IB, and IC and class II (HLA‐DR and HLA‐DQ) antigens (data not shown). We further examined HLA expression in HTCECs by using FACS analysis. In the unstimulated condition, there was constitutive expression of HLA class I but very low expression of class II antigens compared with controls. Results of FACS analysis and median fluorescence index (MFI) values are provided in Figure 4A and 4B. The difference between the MFI of class IA, IB, and IC and that of control was significant (*p* = .003); the difference between the MFI of class II antigens and that of control was not significant (*p* > .05).

After stimulation with IFN‐γ, HTCECs expressed high levels of class I and class II antigens (Fig. 4C). The highest expression was observed for HLA class I and HLA‐DR, followed by lower expression of HLA‐DP and very low expression of HLA‐DQ. After treatment with TNF‐α alone, HTCECs showed low expression for all HLA antigens, with the exception of class I, which showed a slight increase in expression compared with control. When treated with a combination of TNF‐α and IFN‐γ, HTCECs showed HLA expression for all antibodies, which was higher than that observed when cells were treated with TNF‐α alone; however, these levels were still lower than the levels observed when HTCECs were treated with IFN‐γ alone. MFI values are provided in supplemental online Figure 1.

## Discussion

We compared LECs and HTCECs at the transcriptional level and found that both expressed stem cell markers and common limbal markers [6, 7, 8, 9, 10, 11]. CK3, a marker for corneal epithelial differentiation, was robustly expressed in LECs but was low in HTCECs, indicating that HTCECs differentiated poorly in the culture conditions we used. We previously reported that ABCB1 was expressed in both HTCECs and LECs [3]. ABCB1 has been reported to contribute to the SP phenotype of ovarian cancer cells [12]. LECs and HTCECs both expressed CX43, which has previously been reported to be expressed in LECs [13]. By using primers previously reported to detect the three isoforms of ΔNp63 [6], we observed that HTCECs expressed the α isoform (known to be important for LSC proliferation and migration) and the β isoform but lacked expression of the γ isoform; although the latter two isoforms have previously been reported to be expressed in resting LSCs, they become upregulated during limbal cell differentiation [6]. ICC analysis of HTCECs showed that some cells expressed Nanog and ABCB5, and we previously reported that HTCEC SP and NSP express ABCG2, ΔNp63 (the antibody used detected all three isoforms), and Sox2 [4], suggesting that HTCECs contain stem cells. LSPs have been reported to have stem cell characteristics, such as colony formation [3, 14]. HTCEC SP and NSP also formed colonies, but the SP formed bigger colonies.

We previously reported consistent HTCEC SP yields of 0.2%, whereas LEC SP yields varied (0.1%–0.8%) [4]. Donor variability and quality of donor tissues are factors known to influence corneal epithelial outgrowths [15], and these might affect SP yields from tissues.

ABCB5 plays a role in LSC maintenance and corneal wound healing [8]. In our study, HTCECs and LECs expressed ABCB5, whereas HTCEC SP and NSP cells both expressed ABCB5; SP had a higher expression, supporting ABCB5 as an important LSC marker [8].

We showed that HLA class IA, IB, and IC could be detected in unstimulated HTCECs, whereas class II antigens HLA‐DR, HLA‐DP, and HLA‐DQ expressions were low or minimal compared with control. This was similar to findings described for unstimulated human corneal epithelial cultures [16, 17]. HLA class II expression in HTCECs was inducible by proinflammatory cytokines. IFN‐γ in particular upregulated HLA class IA, IB, and IC and class II HLA‐DR, HLA‐DP, and HLA‐DQ expression. Induction of HLA‐DR expression by IFN‐γ stimulation in human corneal epithelial and endothelial cultures has been demonstrated previously [16, 18]. There is limited literature on induction of HLA‐DP in non‐marrow‐derived cells or HLA‐DP‐negative populations. However, our results show that HTCECs mimic the immunogenicity of human corneal epithelium [18], wherein HLA class II (DR and DP) expression was inducible by IFN‐γ treatment, and a low but concomitant HLA‐DQ expression was related to cellular differentiation.

Chemokines are important for immune cell trafficking in pathological and physiological conditions. CXCR4 expression and CXCL12 ligand secretion have previously been reported in the cornea [19, 20]. We showed that HTCECs constitutively express CXCR4 and are chemotactic in response to CXCL12.

## Conclusion

We provide the first data on characterization of ABCB5 in LSPs, supporting the importance of this marker as an LSC marker. Furthermore, the presence of SP cells in the HTCEC cell line that expresses both ABCG2 and ABCB5 supports the use of the SP cell assay as a useful tool for selection of stem cells. SP HTCECs also contained a significant number of CXCR4 positive cells, which may be useful for studying stem cell migration. We also provide evidence that HTCECs are in many ways similar to LECs and are therefore suitable as a robust model for the study of LSC biology.

## Author Contributions

B.S.: conception and design, collection and/or assembly of data, manuscript writing; S. Ahmad, S. Ali: conception and design; N.M.L.: collection and assembly of data; A.M.: conception and design, collection and/or assembly of data, manuscript writing, final approval of manuscript.

## Disclosure of Potential Conflicts of Interest

The authors indicated no potential conflicts of interest.

## Supporting information

Supporting Information
