# Supplementary material for: A Human Corneal Epithelial Cell Line Model for Limbal Stem Cell Biology and Limbal Immunobiology
Source: Stem Cells Transl Med. 2016 Oct 14;6(3):761–6. doi: 10.5966/sctm.2016-0175 (PMC5442771; doi:10.5966/sctm.2016-0175)
Supplement: Supplementary file 1 — Supporting Information [file SCT3-6-0761-s001.pdf]

Supplemental Information – Meeson et al.

Supplemental Figure

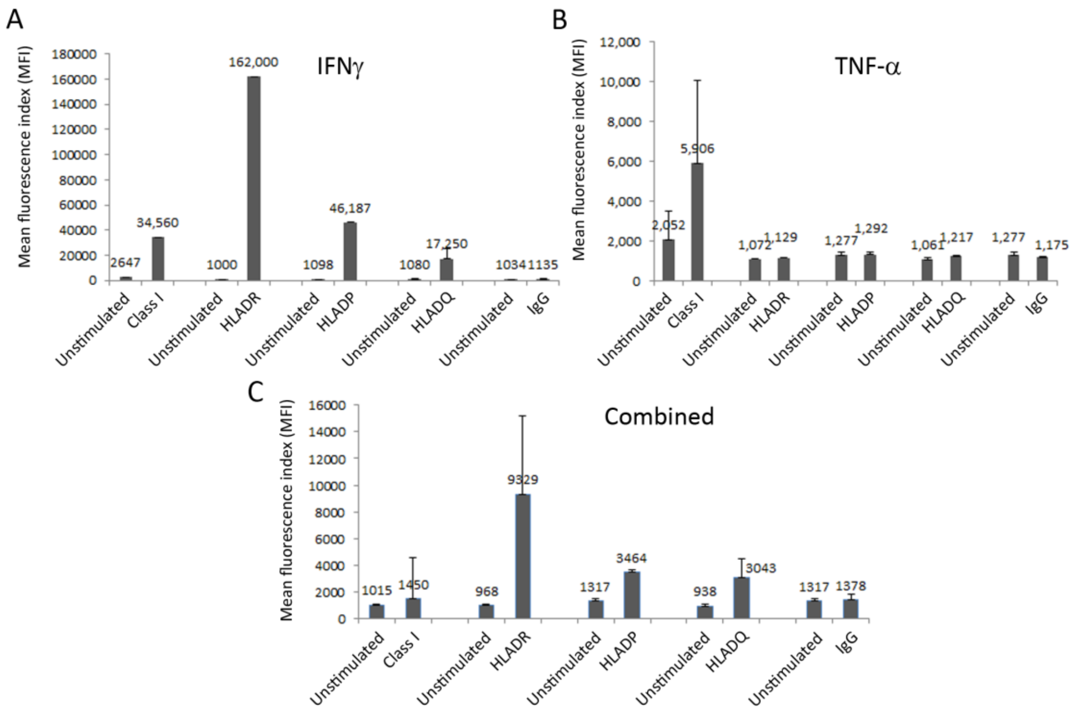

Figure S1. Median Fluorescence Index of HLA Class I-A,B,C and Class II expression in HTCEC for control (unstimulated) and stimulated populations under cytokines treatment at 10 ng/ml for 3 days (N=3). Treatment by (A) Interferon-γ alone (B) Tumour necrosis-α alone(C) combined treatment. Results are from two biological replicates.

Supplemental Tables

Table S1. Antibodies used in flow cytometry and ICC.

Antibody	Dilution	Supplier
PRIMARY		
Mouse monoclonal FITC anti-human HLA-DR (Clone L243)	1:20	Biolegend
Mouse monoclonal FITC anti-human HLA-A,B,C (Clone W 6/32)	1:20	Biolegend
Mouse FITC anti-human HLA-DQ (Clone HLADQ1)	1:20	Biolegend
Mouse monoclonal Anti-human HLA-DP (Clone HI43)	1:20	Abd Serotec
Mouse monoclonal anti-human CXCR4 (clone 44716.111)	1:100	R & D Systems
Mouse monoclonal anti-human ABCB5 (clone 5H3C6)	1:100	Abcam
Rabbit polyclonal anti-human ABCG2	1:100	Novus Biologicals
Mouse monoclonal anti-human NANOG (NNG-811)	1:100	Abcam
SECONDARY		
Goat anti-mouse FITC-conjugated IgG	1:25	Jackson Immunology Research Laboratories
Goat anti-rabbit FITC-conjugated IgG	1:25	Jackson Immunology Research Laborotories

Table S2. Oligonucleotides primers and amplification conditions.

Gene	Primers	Sequences	Product Length (bp)	Amplification Cycles	Annealing temperature °C
LIMBAL STEM CELL MARKERS					
p63			143	35	55
	Forward	GTGATGATGGTTCACGTTGG			
	Reverse	ACATGACGTCGGGTGTTTTT			
CK3			145	35	53
	Forward	GGATGTGGACAGTGCCTATATG			
	Reverse	AGATAGCTCAGCGTCGTAGAG			
ABCG2			143	35	55
	Forward	GCGTGCTGTGCCCACTCAAA			
	Reverse	AGCATGTGCACGGTGCGTTC			
C/EBPδ			111	35	55
	Forward	ACTCAGCAACGACCCATACC			
	Reverse	CGCTCCTATGTCCCAAGAAA			
Connexin 43			249	35	53.4
	Forward	ATGAGCAGTCTGCCTTTCGT			
	Reverse	TCTGCTTCAAGTGCATGTCC			
Bmi-1			132	35	51.7
	Forward	CTGGAGAAGGAATGGTCCAC			
	Reverse	GCCTTGTCACTCCCAGAGTC			
ABCB5* Hs 02889060_m1		N/A	98	35	55
GAPDH			100	35	55
	Forward	ATG GGG AAG GTG AAG GTC G			
	Reverse	TAA AAG CAG CCC TGG TGA CC			
STEM CELL ANTIGENS					
Sox2	Forward	AAC CCC AAG ATG CAC AAC TC	100	35	54
	Reverse	GCT TAG CCT CGT CGA TGA AC			
Oct 4					
	Forward	AGT GAG AGG CAA CCT GGA GA	110	30	54
	Reverse	ACA CTC GGA CCA CAT CCT TC			
NANOG	Forward	TTC CTT CCT CCA TGG ATC TG	213	35	53.1
	Reverse	TCT GCT GGA GGC TGA GGT AT			

**Taqman probe [Applied Biosystems].*

Table S3. Statistics for image quantification analysis (signal intensities) of CXCR4 and ABCB5 expression in limbal SP and NSP in HTCEC.

	N	Mean signal intensity	± SD	*P value
CXCR4				
- SP	46	18.16	7.21	0.00
- NSP	40	8.90	3.48	
ABCB5				
- SP	160	26.34	8.70	0.02
- NSP	160	24.16	8.07	

*Independent t-test.
